# Geometry-Driven Cell Organization Determines Tissue Growths in Scaffold Pores: Consequences for Fibronectin Organization

**DOI:** 10.1371/journal.pone.0073545

**Published:** 2013-09-05

**Authors:** Pascal Joly, Georg N. Duda, Martin Schöne, Petra B. Welzel, Uwe Freudenberg, Carsten Werner, Ansgar Petersen

**Affiliations:** 1 Julius Wolff Institute, Charité - Universitätsmedizin Berlin, Berlin, Germany; 2 Berlin-Brandenburg School for Regenerative Therapies, Charité - Universitätsmedizin Berlin, Berlin, Germany; 3 Berlin-Brandenburg Center for Regenerative Therapies, Charité - Universitätsmedizin Berlin, Berlin, Germany; 4 Center for Musculoskeletal Surgery, Charité - Universitätsmedizin Berlin, Berlin, Germany; 5 Max Bergmann Center of Biomaterials Dresden, Leibniz Institute of Polymer Research Dresden, Dresden, Germany; 6 Center for Regenerative Therapies Dresden, Technische Universität Dresden, Dresden, Germany; Université de Technologie de Compiègne, France

## Abstract

To heal tissue defects, cells have to bridge gaps and generate new extracellular matrix (ECM). Macroporous scaffolds are frequently used to support the process of defect filling and thus foster tissue regeneration. Such biomaterials contain micro-voids (pores) that the cells fill with their own ECM over time. There is only limited knowledge on how pore geometry influences cell organization and matrix production, even though it is highly relevant for scaffold design. This study hypothesized that 1) a simple geometric description predicts cellular organization during pore filling at the cell level and that 2) pore closure results in a reorganization of ECM. Scaffolds with a broad distribution of pore sizes (macroporous starPEG-heparin cryogel) were used as a model system and seeded with primary fibroblasts. The strategies of cells to fill pores could be explained by a simple geometrical model considering cells as tensioned chords. The model matched qualitatively as well as quantitatively by means of cell number vs. open cross-sectional area for all pore sizes. The correlation between ECM location and cell position was higher when the pores were not filled with tissue (Pearson’s coefficient ρ = 0.45±0.01) and reduced once the pores were closed (ρ = 0.26±0.04) indicating a reorganization of the cell/ECM network. Scaffold pore size directed the time required for pore closure and furthermore impacted the organization of the fibronectin matrix. Understanding how cells fill micro-voids will help to design biomaterial scaffolds that support the endogenous healing process and thus allow a fast filling of tissue defects.

## Introduction

During the last 60 years, the design of biomaterials has evolved from bioinert (first generation) to bioactive and biodegradable materials (second generation) and finally to materials that will stimulate a cell specific response at the molecular level (third generation) [1], [2]. This last generation aims at stimulating the cells, supporting them in their task to regenerate and at enabling them to accomplish healing in a fast and effective way. From endogenous mechanisms of bone healing, a tissue with the capacity to completely restore its function and structure, it is known that fibroblasts are among the first cells to enter the regenerative scenery. For healing of e.g. bone defects, matrix formation by fibroblasts paves the way for more complex tissue formation and maturation [3]. In order to form an organized extracellular matrix structure, it is necessary for the cells to cooperate and communicate by using a combination of their “sense of smell” (biochemical cues) but also their “sense of touch” (mechanical cues) [4]. 2D environments lack topological information present in the initial ECM tissue samples and therefore limit the cell possibility for 3D matrix adhesion, more specifically, spatial distribution of receptors anchored to the ECM [5], [6]. Indeed, in 2D and 3D environments, cells will exhibit differences in terms of morphology, migration and signaling with their behavior strongly relating to the kind of 3D conditions provided [7]. Therefore, in the case of in-vitro experiments, three-dimensional matrices are recognized to be closer to physiological conditions. In scaffold design, substrate properties such as hydrophilicity have been shown to correlate with the capacity of the cells to adhere and to spread on pore walls [8]. Stiffness [9], pore size [10] as well as geometry [11] have also been gaining more attention lately as they have been shown to influence cell behavior. Local geometry was shown to have an impact on cell growth and viability by controlling the ability of the cell to expand [12] as well as a different number of cell functions [13]. However, larger scale geometry has also been observed to have an influence on tissue growth [11].

Macroporous scaffolds have the advantage of an enhanced nutrient supply to the cells and removal of metabolic waste out of the scaffold [14] due to convective transport through the pores. They also promote cell migration when compared to nanoporous hydrogels. Hypoxia, nutrient transport as well as cell spreading are constant issues in the latter as the cells not only have to actively degrade their environment before they are able to migrate or spread to their full size but diffusion is hindered due to their nanoporous structure.

At the early stage of dermal wound healing, the existing matrix is mainly composed of fibronectin, type I and type III collagen as well as fibrin/fibrinogen and proteoglycans [3], [15]. Early fibronectin deposition is essential as polymerization of type I and type III collagen depends on fibronectin [16] which will then further modulate the cell response to its environment [17]. Inhibition of fibronectin polymerization has also been linked with an increase in levels of endocytosed collagen I [18]. The localization of fibronectin within a scaffold will affect the localization of the collagen deposition.

In this work, we hypothesized that a simple geometrical model adapted from Bidan et al. [11] could accurately predict the number of cells involved in the process of pore filling in a macroporous scaffold and that the event of pore closure would impact the ECM distribution. Macroporous starPEG-heparin cryogels provide a broad range of pore sizes. Such scaffolds, used as a model system, offer human primary dermal fibroblasts a broad spectrum of voids to be filled. Cell spreading and subsequently pore filling by proliferation and production of the extracellular matrix component fibronectin was investigated in a timeframe between one and seven days. Pore filling was analyzed by immunostainings and confocal/multiphoton microscopy together with a custom made Matlab based code to gain information about cell location and fibronectin production. Time dependent changes in the open cross-sectional area of the pores were then compared to the simple geometrical model of pore filling. The aim of this study was to understand cell organization inside macroporous scaffolds during pore filling as well as its impact on ECM (here fibronectin) distribution. Such an understanding is fundamental for the design of biomaterial scaffolds controlling tissue growth for regenerative purposes.

## Materials and Methods

### Ethics Statement

Primary dermal human fibroblasts were used for this study. Ethical approval was obtained from the ethics committee of Charité – Universitätsmedizin Berlin and donor’s written informed consent was given.

### Macroporous starPEG-heparin Cryogel Scaffolds

The fabrication of well defined starPEG-heparin cryogels with large interconnected macropores suitable for the ingrowth of mammalian cells was already described elsewhere [19]. Briefly, we combined the well-established network formation via chemical crosslinking (EDC/sulfo-NHS chemistry) of amino terminated starPEG and heparin [20], [21], [22] with the cryogelation technology [23].

The reaction mixture containing the gel precursors and the activation agents was prepared as previously reported in [19], [22] and pipetted into the cavities of a 96-well plate. A molar ratio of starPEG to heparin of γ = 3 and the total precursor concentration (starPEG and heparin) of 11.6% (w/w) was used for the present study. After freezing at −20°C overnight, the samples were lyophilized for 24 hours. The resulting dry cylindrical cryogel scaffolds were cut into discs, which were punched to the desired diameter by means of a punching tool (Hoffmann GmbH, Qualitätswerkzeuge, München, Germany). The scaffolds used in the present study were about 3 mm in height and 5 mm in diameter after swelling in PBS (phosphate buffered saline, pH 7.4).

Prior to seeding cells, cryogel discs were sterilized with UV-light for 1 hour. To improve cell adhesion, the starPEG-heparin hydrogel matrix was biofunctionalized with an RGD containing peptide (cyclo(Arg-Gly-Asp-D-Tyr-Lys), Peptides International, Louisville, KY, USA) as described in [19]. Briefly, heparin carboxylic acid groups of swollen cryogels were activated with s-NHS/EDC solution (25 mM s-NHS, 50 mM EDC in 67 mM phosphate buffer (pH 5)) for 60 min at 4°C followed by flushing in borate buffer (100 mM, pH 8.0, 4°C). Next, the scaffolds were incubated in RGD-solution (200 µg/ml; dissolved in 100 mM borate buffer, pH 8) for 3 hours at room temperature. Finally, all samples were washed in PBS three times.

StarPEG-heparin cryogels were analyzed for mechanical properties, swelling, and 3-D architecture as reported elsewhere [19].

### Cells and Culture Medium

Primary dermal human fibroblasts isolated from skin samples were used at passage 4–6. Cells were expanded in culture medium consisting of Dulbecco’s modified Eagle’s medium (DMEM, Invitrogen) supplemented with 10 vol-% fetal bovine serum (# S 0115, Biochrom AG), 1 vol-% penicillin/streptomycin (# A 2213, Biochrom AG) and 1 vol-% nonessential amino acids (# K 0293, Biochrom AG) in a humid incubator with 5% CO_2_. Additionally ascorbic acid in a final concentration of 1.36 mM was added to the medium for cell seeded constructs to enhance ECM formation. Indeed, ascorbic acid has been shown to influence dermal fibroblasts proliferation and increase in collagen secretion. Other studies using trabecular meshwork cells also suggest an effect of ascorbic acid on fibronectin and laminin [24], [25].

### Cell Seeding into Scaffolds

Cells were trypsinized and brought into suspension in a concentration of 7.5×10^3^ cells/µl in culture medium. The high seeding concentration was chosen to drive cells into pore filling without a prolonged phase of proliferation. The latter might itself be influenced by pores size and thus make the starting situation inhomogeneous. The actual concentration was chosen to result in a cell density that is close to the saturation cell density on the pore wall determined in preliminary seeding experiments. Scaffolds were placed on sterilized filter papers for 20 seconds to remove excess PBS from the pores. Scaffolds were dipped into the cell suspension, which they immediately absorbed. They were subsequently transferred into a 12-well culture plate without additional medium. Cell seeded constructs were kept in the humid incubator for 60 min to allow cell adhesion. Cell constructs were subsequently washed in culture medium to remove unattached cells.

### Immunostainings

At the end of each experiment, cell-seeded constructs were fixed in 4% paraformaldehyde (PFA) at 4°C overnight. Then, they were stained for actin filaments (Alexa fluor 488 Phalloidin, # A12379, Invitrogen), fibronectin (anti-fibronectin antibody, # ab23750, Abcam plc) and cell nuclei (DAPI, # D3571, Invitrogen). Briefly, for confocal microscopy, scaffolds were washed in PBS three times for 5 minutes. Permeabilization was then performed using 0.2% Triton-X100 in PBS at room temperature. After washing the scaffolds twice in PBS for 5 minutes, blocking in 1%BSA/PBS at room temperature for 30 minutes was realized. Scaffolds were then incubated overnight at 4°C with primary antibody for fibronectin in Dako antibody diluent (dilution 1∶150, # S3022, Dako). Subsequently, washing in 0.2% Triton-X100 in PBS at room temperature was realized four times for 5 min. Scaffolds were then incubated for one hour in an incubation chamber at room temperature in 2% normal serum Donkey and 1% BSA in TBS with the Cy3 conjugated Anti-Rabbit together with Phalloidin Alexa 488. Subsequently, three washing steps in 0.2% Triton-X100 in PBS for 5 minutes were performed, as well as one washing step in distilled water for 3 min. Scaffolds were then stained for cell nuclei by DAPI (1∶1500) in distilled water for 5 min at room temperature. Two final washing steps were performed in distilled water for 3 minutes.

### Imaging

All images were taken using a LEICA SP5 confocal microscope equipped with a Mai Tai HP multiphoton laser and a 25× water immersion objective. Emitted light from the samples was detected using two non descanned detectors (NDD) as well as internal photomultiplier detector (PMT) between 555 nm and 625 nm to detect Cy3 secondary antibody emission with a resolution of 512×512 pixels. For the 20 µm z-stack, 6 slices were recorded resulting in voxel dimensions of z = 4 µm and x = y = 400 to 700 nm depending on the size of the region of interest. A HCX IRAPO L 25.0×0.95 water objective was used having a numerical aperture of 0.95. Laser power and detectors parameters were kept constant throughout the imaging of the different pores. Twelve pores out of two scaffolds were analyzed for each time point.

### Analysis of Cell Location, Maximal Spreading Length and Minimum Pore Wall to Wall Distance

Acquired images were analyzed using a Matlab based code (Supporting information S1) which allowed the loading of a 20 µm z-stack, contouring the pore for each z-stack picture, selection of nuclei position through the z-stack as well as marking the points of connection between cells and scaffold. Maximal spreading length *d*_cell,spread_ of each cell was then extracted as well as closest distance between the wall of the pore and the cell nucleus *d*_nucleus-wall_. Maximal spreading length mean value d*_cell,spread_ was then calculated. From the drawing of the individual pore contours, an elliptic fit was performed and wall to wall distance *d*_pore,min_ was defined as the small axis of the elliptical fit. The distributions of *d*_cell,spread_ and *d*_pore,min_ were used to define different categories of pore sizes: small, medium and large.

### Analysis of Actin and Fibronectin within Pores and their Correlation

From the elliptic fit that was obtained from each of the contours drawn, a 360 degrees profile plot of 200 points resolution of intensity between the pore wall and the center of the elliptic fit was obtained in steps of two degrees. For both green (actin) and red (fibronectin) channels, an average over the z-stack was plotted to get an indication of actin and fibronectin distribution in the pores. Also, to study the colocalization between actin and fibronectin, the Pearson correlation coefficient was extracted from the maximum projection of the z-stack after having drawn the pore contour using the software Volocity (PerkinElmer) to define the region of interest. Developed by Manders et al. in 1992, the Pearson correlation coefficient aims at measuring the strength of linear relationship between two fluorescent intensities images. It is based on the comparison between pixel intensity of the same coordinates of different channels and yield values between 1 (perfect correlation) and −1, with 0 representing a random distribution [26], [27]. The statistical analysis performed to compare the different groups was done using a paired samples T-Test.

### Open Cross-sectional Area of the Pore

The fibronectin channel from the z-stack was used to get an idea of how fast and effective the filling was for the different categories. The projections of the z-stack were used to calculate the remaining empty area within the pore. A ratio was then made between the space to be filled (i.e. remaining open cross-sectional area) and the initial cross-sectional area of the pores and plotted as a function of the number of cells within that pore for the different pore size categories and time.

### Modeling of Pore Closure by Cell Proliferation

To model the pore filling behavior of the fibroblasts, the principle of the chord model [11] was used. Initially the pore was modeled as a 2D elliptical area, which was filled step-by-step by another cell. Each cell was modeled as a line attached at two points to the current border. The area framed by the cell and the border was assumed as filled by the cell and the line was set as new border. The different cell positions available and the corresponding covered areas were then calculated. From this spectrum of potentially covered areas a certain range was selected (changing the lower cut-off point) represented by a factor R between R = 0 (full spectrum) and R = 1 (only the maximum area of the spectrum). The area covered by the cell and the according cell position within this variation range were then picked randomly. The simulation was stopped once the cells had filled the pore area.

The input parameters for the simulation were measured from the experiments and were the initial area A, the average elliptical ratio Φ_ellips_, and the range of cell spreading length d*_cell,spread_ ± σ*_d_*_cell,spread_. The elliptical fit enabled the calculation of the elliptical ratio of each pore (Φ_ellips_ = length of long axis/length of short axis). For each step, *d*_cell,spread_ was chosen randomly in the range of cell spreading length observed (d*_cell,spread_ ± σ*_d_*_ cell,spread_).

## Results

### Key Characteristics of the starPEG-heparin Cryogel Scaffolds

Subzero temperature treatment of the gel forming reaction mixtures and subsequent lyophilization resulted in macroporous starPEG-heparin scaffolds as previously reported in [19]: the spongy materials exhibit a porosity of about 90% and undergo rapid swelling in aqueous solution. The swollen bulk materials are soft (bulk Younǵs modulus about 6 kPa [19]) but very tough as demonstrated by uniaxial compression experiments [19]. They consist of interconnected irregular macropores surrounded by rather low hydrated polymer regions (pore walls) of only about 10 µm in width. Due to their high polymer concentration (cryoconcentration effect [23]) the pore walls exhibit a microscale Younǵs modulus of about 1500 kPa [Welzel/Friedrichs, in prep.]. For a more detailed discussion of the cryogel properties the reader is referred to [19] and [Welzel/Friedrichs, in prep].

### Cell Maximum Spreading Length

**Figure 1:**
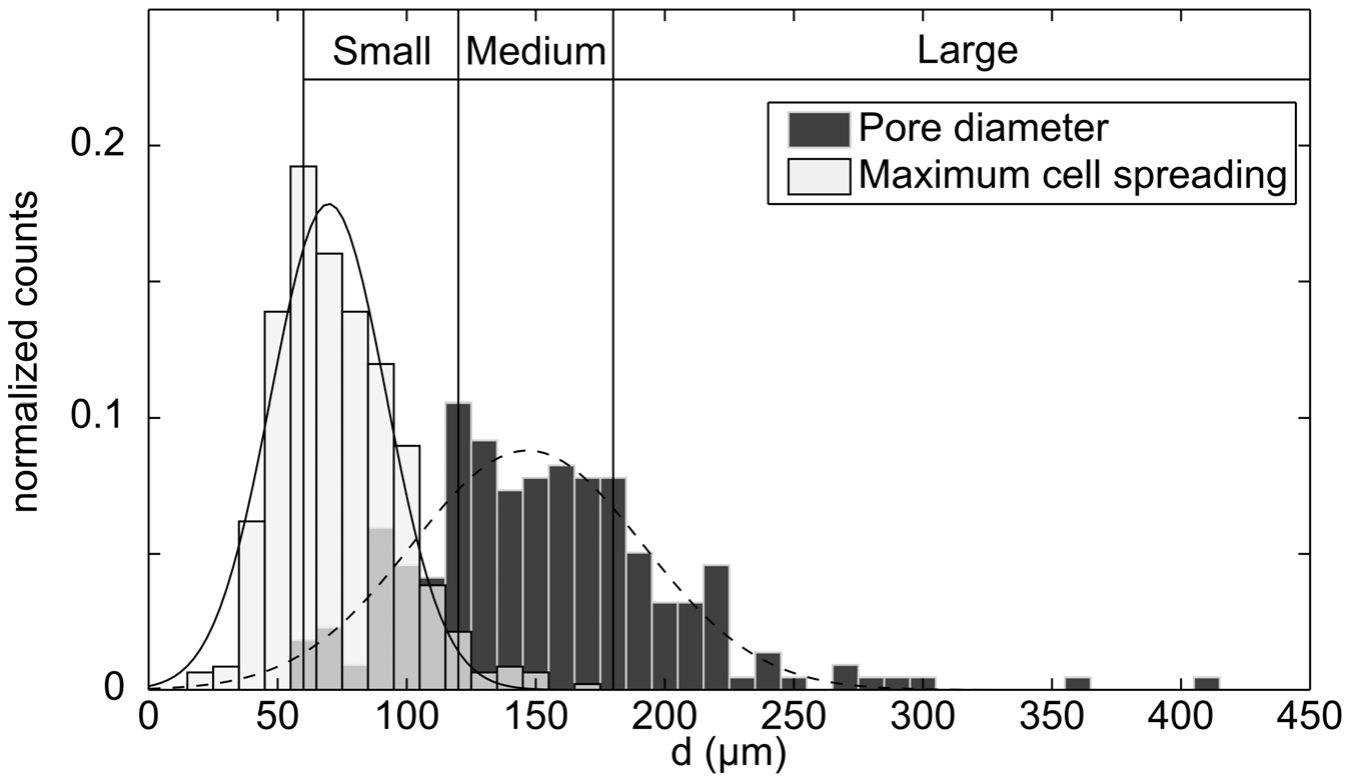
Maximal spreading length of fibroblasts and pore wall to wall distance within StarPEG-heparin cryogel. Maximal spreading length *d*_cell,spread_ of the different human dermal fibroblasts are represented by white bars and wall to wall distance *d*_pore,min_ of the pore analyzed in the scaffold by dark grey bars. The overlapping area is displayed in light grey. Gaussian fit was performed for maximum cell spreading (continuous line) and pore diameter (dashed line) distribution. The data was obtained by combining all the imaging performed on the starPEG-heparin cryogel (N_pores_ >100) and cells contained in pores (N_cells_ >200).”

The maximal spreading length of the cells within the scaffold as well as the minimum wall to wall distance of the pores *d*_pore,min_ analyzed in the scaffold are shown in Figure 1. Fibroblasts had a maximal spreading length of *d_cell,spread_* = 73.6±22.7 µm. The distributions of *d*_cell,spread_ and *d*_pore,min_ were used to define different categories of pore sizes: Small pores (60< *d*_pore,min_ <120 µm), where most cells measured had the possibility to spread entirely across the pores. Medium pores (120< *d*_pore,min_ <180 µm) where only a marginal number of cells (3.5%) were measured to be able to reach such a size. Large pores were defined by *d*_pore,min_ >180 µm where no cell was observed to cross the pores at day 1.

### Cell Spreading Behavior Inside Pores and Pore Filling

**Figure 2:**
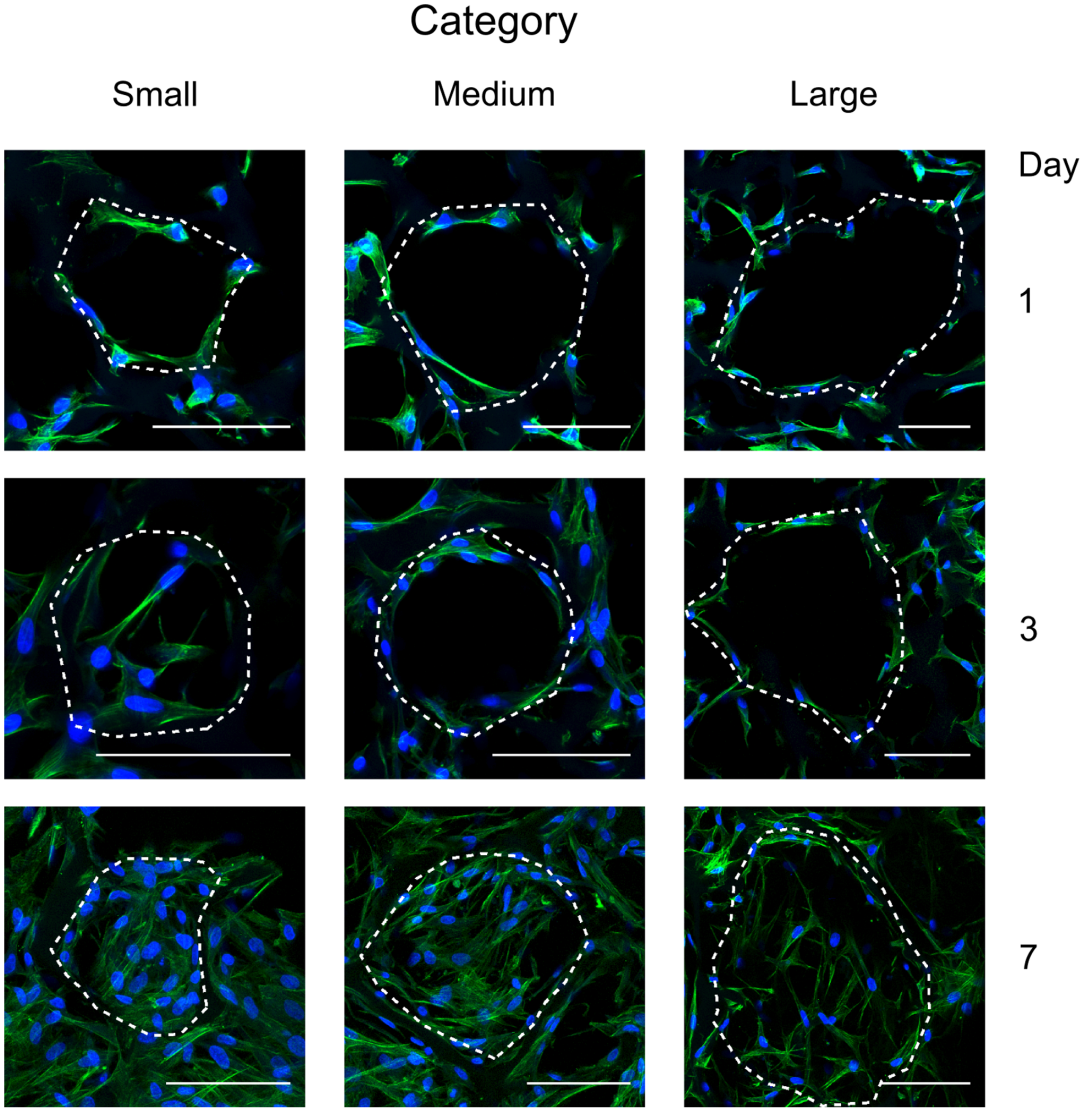
Filling of scaffold pores by fibroblasts for three pore size categories and three time points. Confocal microscopy for visualization of actin cytoskeleton (green) and cell nuclei (blue) to illustrate the filling of scaffold pores by human fibroblasts at day 1, 3, and 7 for the three pore size categories (scale bar equals 100 µm). Data shown corresponds to the maximum projection images of the 3D image stacks recorded inside scaffolds. During the imaging, scaffold pores could be identified due to background staining of the scaffold material and are highlighted by dashed lines.

Figure 2 provides an insight into the general behavior of the pore filling process by human fibroblasts depending on incubation time and the scaffold pore size. As mentioned above, three categories were distinguished: small, medium and large pores. After 24 hours, human fibroblasts were fully spread on the scaffold walls. After 3 days, fibroblasts were observed to spread across the pores. Full pore filling was not observed in the medium and large pore size categories at day 3. After 7 days, all pores were closed and completely filled with cells.

**Figure 3:**
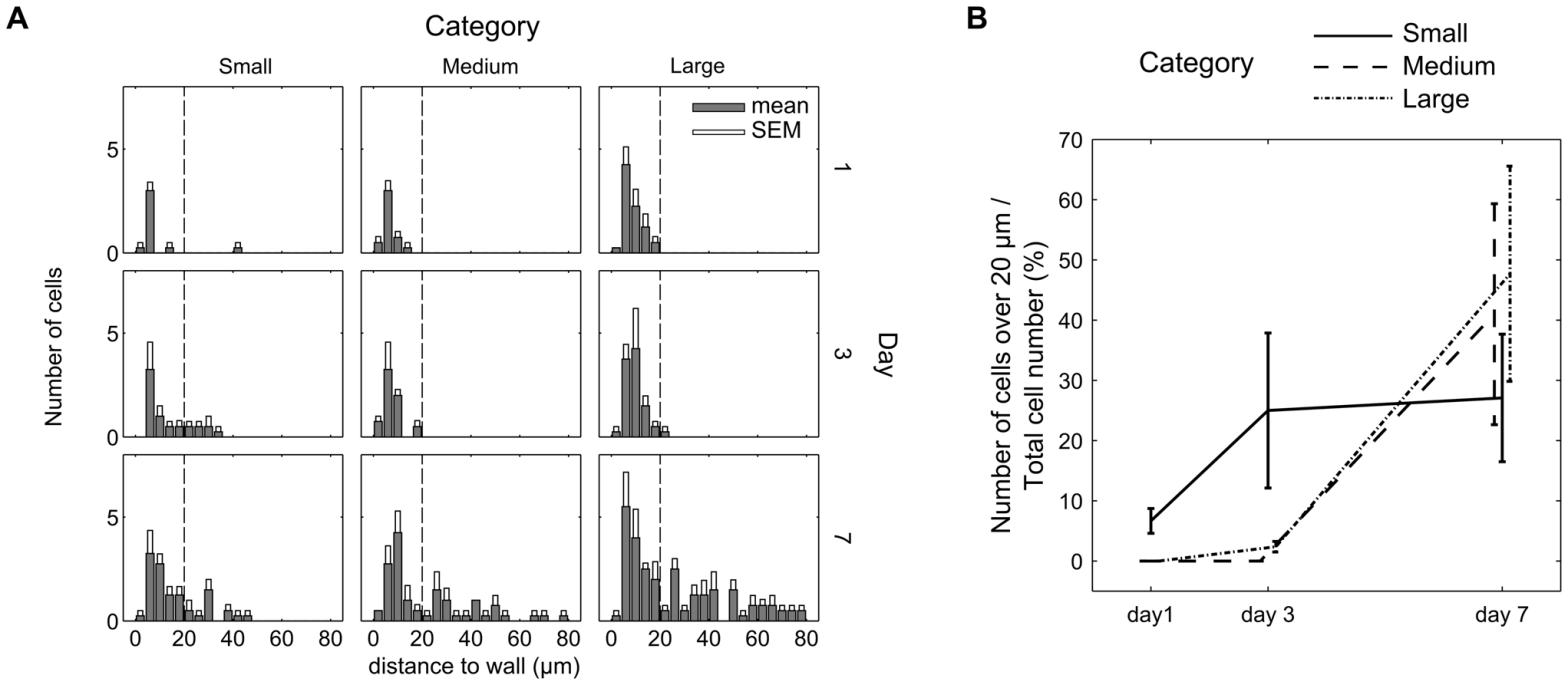
Quantification of cell number during pore filling. Evolution of cell position (cell nuclei) as a function of time and pore size for human fibroblasts (mean values of n_pores_ = 4 for each category, SEM = standard error of the mean) (a), the derived ratio between the number of cells that are located within the pores (#cells_in_pore_), and the total number of cells (#cells_total_) in the pore (b).

The quantification of *d*_nucleus-wall_ is shown in Figure 3a. The vertical line at 20 µm indicates an arbitrary limit corresponding to cells located primarily on the wall of the scaffold pores. This limit was chosen due to the fact that, at day 1, almost all cells were located on the wall and data analysis of nuclei position gave values of *d*_nucleus-wall_ <20 µm. Figure 3b displays how the ratio of the cells having crossed the 20 µm limit changed over time in the three categories. As seen in the histology results, this quantitative data indicates that most cells were located on the walls of the pores at day 1. After 3 days, the cells were able to migrate or stretch further away from the wall and spread across the pores in the small category size. Interestingly, even if the cells have the possibility to fully cross over the pore in the small category, most of the cells were observed to be on the wall. Between 3 and 7 days, for the smaller pore, even though the total cell number was still increased, the ratio of cells more than 20 µm away from the wall divided by the total cell number showed a saturation behavior at a value of #cells_in_pore_/#cells_total_ ≈ 0.3. For pores of medium and large category, #cells_in_pore_/#cells_total_ increased strongly from day 3 to day 7 from 0.0 up to 0.4 and 0.5 respectively indicating a delayed pore closure compared to the small pores.

### Pore Remaining Area

**Figure 4:**
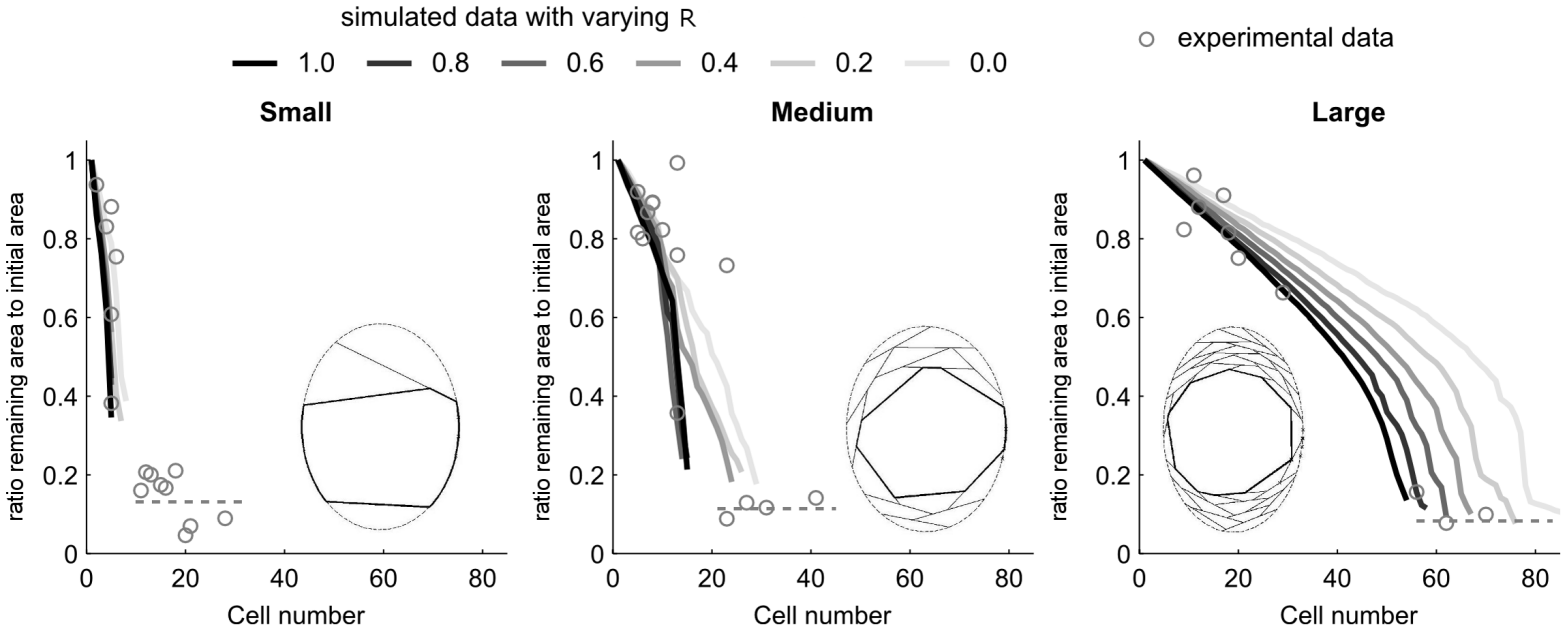
Remaining open cross-sectional area as a function of the number of cells. The ratio between the space to be filled (i.e. remaining open cross-sectional area) and the initial cross-sectional area of the pores was plotted as a function of the number of cells within that pore for the different categories. Experimental data is shown as circles for the different categories. Simulation data is displayed as lines with different shadings from black to light grey for different variation ranges for the covered area (R = 1: new cells positioned at locations of largest covered area per cell; R = 0: random position of new cells on the remaining inner pore surface). Simulation was based on a 2D model. Experimental data points were extracted from z-stack maximum projection for 2D analysis. Lines represent the average of ten simulations. Simulation stopped when the remaining inner diameter of the pore was smaller than the cell size. Within each graph the cell organization for a remaining area between 0.5 and 0.6 of the initial pore area according to the simulation is given. Dashed lines represent pore closure extracted from experimental data. Due to the porosity of the fibronectin network the remaining open area did not reach a zero value.

The simulation of the filling process illustrates the dependency of the open cross-sectional area on the total number of cells and gave an indication of how many cells were required to fill the pore to a certain degree (Fig. 4).

For the modeling, the initial mean cross-sectional area of the pores (area = 10.3, 22.5 and 44.9×10^3^ µm^2^ for small, medium and large categories, respectively) and d*_cell,spread_ served as input parameters (both parameters were measured from experiments). The pore cross-sectional geometry was simplified to an elliptic shape taking into account the average elliptic ratios Φ_ellips_ = length of long axis/length of short axis for the different pore size categories (Φ_ellips_ = 1.31, 1.29 and 1.47 for small, medium and large categories, respectively). A variety of simulations on the same geometry with different values for the random factor R were performed. Comparison between experimental data and simulation suggested a trend towards cells being positioned to cover a large area rather than taking a random position.

### Fibronectin Formation Inside Scaffold Pores

**Figure 5:**
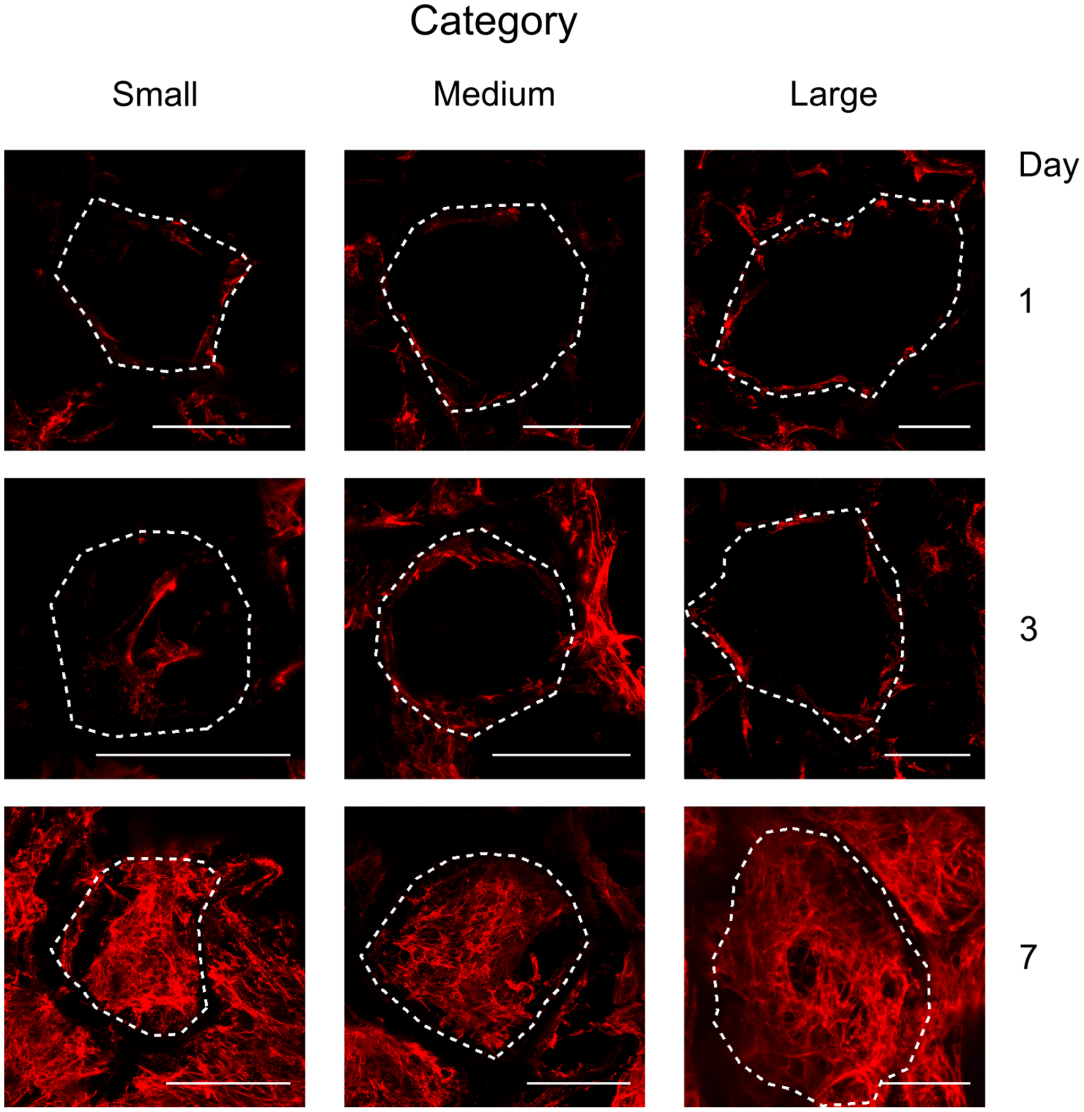
Fibronectin organization for three pore size categories and three time points. Confocal microscopy for visualization of fibronectin network (red) to illustrate the filling of scaffold pores until day 1, 3, and 7 and the three pore size categories (scale bar equals 100 µm). Data shown corresponds to the maximum projection images of the 3D image stacks recorded inside scaffolds. During the imaging, scaffold pores were observed due to some background staining and where delimited here by dashed lines.

Figure 5 shows fibronectin staining additional to the DAPI and actin staining from figure 2 to illustrate how the early ECM is deposited in the scaffold pores as a function of time and pore size category. At day 1, fibronectin was observed only at discrete locations on the walls of the pores. No individual fibers and no network formation could be identified. After 3 days, fibronectin had been deposited by the cells in a fibrillar form and the comparison with the actin cytoskeleton location indicates that its distribution followed cellular organization: fibers of fibronectin were observed across the pores only in the small category size. After 3 days, a higher amount of fibronectin fibrils were visible. This was potentially due to an additional recruitment of fibronectin by the cells through FN - integrin interaction and subsequent conformational changes during polymerization as reported in the literature [28]. After 7 days, fibrillar fibronectin was observed to fill pores of all size categories and was visible across the pores as well as on the pore walls forming a dense network.

**Figure 6:**
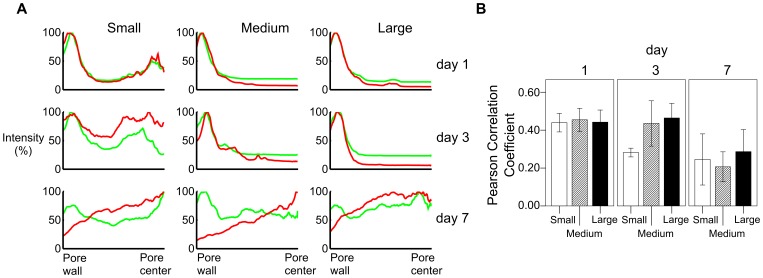
Distribution of fibronectin and actin inside scaffold pores. Actin (green) and fibronectin (red) intensity distribution from the pore center to the pore wall as extracted from histological images (Fig. 6a, n_pores_ = 4 for each category). From the maximum projection of the z-stack and the contoured pore, the Pearson coefficient for green and red channel colocalization was calculated. The whole intensity data range for each channel (0–255) was considered here (Fig. 6b, n = 5 for each category).

To quantify cell and ECM distribution the average channel intensity for the green channel (actin) and the red channel (fibronectin) were analyzed as shown in Figure 6a. An overlayed intensity profile was plotted representing the radial distribution from the pore wall to the center of the pore (for details see method section). At day 1, a high peak was observed in the close vicinity of the walls for all pore size categories indicating that actin and fibronectin, if any, were primarily located on the surface of the pores. Only for small pores an additional peak was observed closer to the pore center. In this case, cell crossing over the pores had already started in some pores accompanied by fibronectin deposition.

After 3 days, for all categories, the main peak for actin remained located next to the pore wall, indicating that most of the cells were still on the pore wall. For the small category pore size, the peak for actin and fibronectin at the pore center had grown strongly: the pore was being filled with cells and ECM. For the medium and large size category, actin and fibronectin were still mainly located next to the wall.

**Figure 7:**
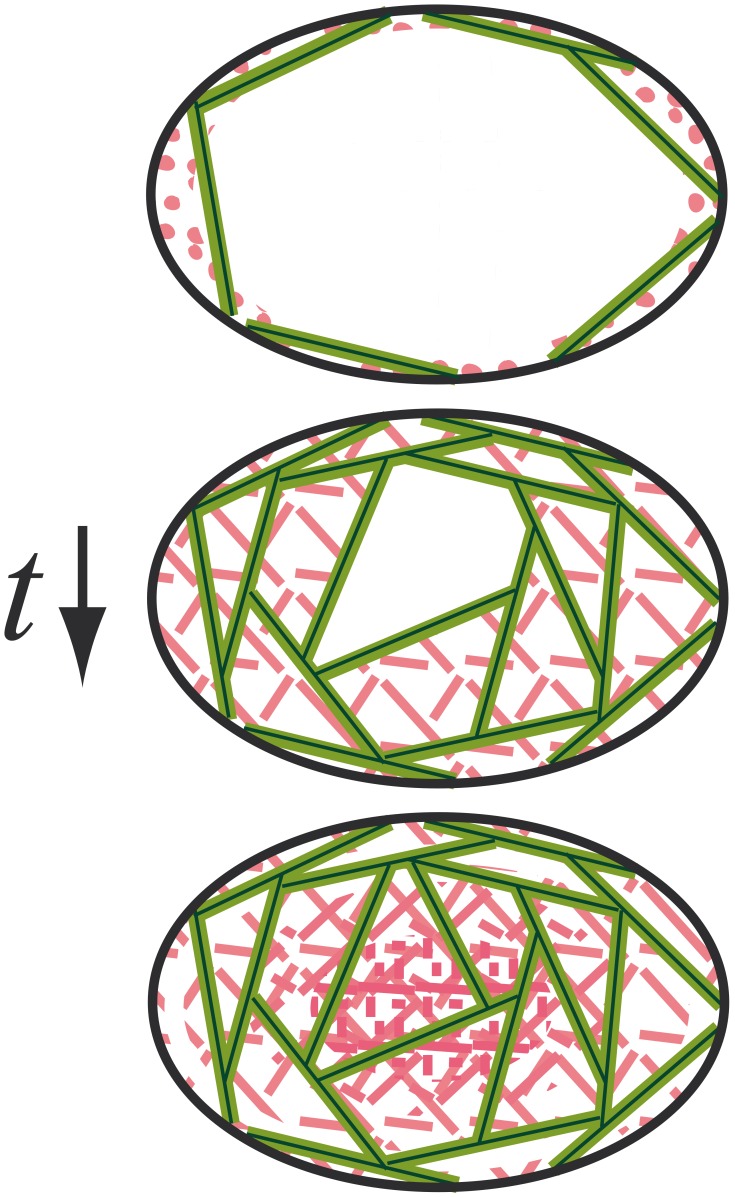
Schematic representation of pore filling as a function of time. Cells are represented by tensioned chords in green and fibronectin is in red. Initially cells are spreading on the pore wall and fibronectin is being produced but not visible as a connected network yet by the cells (top). The pore is subsequently filled by cells with time according to the model and produce fibronectin fibers being unfolded due to cell forces (middle). Finally the pore closes completely and at the same time fibronectin is further compacted towards the center (bottom).

The intensity distribution for day 7 indicated an almost homogeneous distribution of cellular actin inside the pores. This was confirmed by the histological images (Figure 2). For fibronectin, the curves showed higher intensity values in the middle of the pores indicating that a denser network had formed in the center of the pores. To further analyze this observation, the colocalization between actin and fibronectin was quantified by calculating the Pearson coefficient. The results for each category and points in time are shown in Fig. 6b. Values were observed to be comparable for the different categories at day 1. At day 3, a drop in correlation value was observed only in the small category, coinciding with the filling of the pore by fibronectin and actin, indicating that cells did not stay located to the place where they produced the fibronectin. This drop was not seen for medium and large categories at day 3. However, a clear drop in the Pearson coefficient was observed at day 7 for both categories. This is interesting since it is the point in time when the pores had been filled with cells and fibronectin comparable to the situation at day 3 for the small pore category. By putting together those groups i.e. group A where the filling of the pore has not occurred (i.e. day 1 for all categories and day 3 for medium and large) and group B where the pore filling has happened (i.e. day 3 for small pores and day 7 for all categories) a significant difference (p<0.05) was observed between PearsonCoeff _group A_ = 0.45±0.01 and PearsonCoeff _group B_ = 0.26±0.04. Fibronectin and actin are only located on the pore wall when the pore is not yet filled. However, once the pore is filled (group B), although fibronectin still seem to be present in the pore wall vicinity, it appears that a change in distribution has occurred, as more fibronectin was observed in the middle of the pores. This process is summarized in figure 7.

## Discussion

The goals of this study were to 1) qualitatively and quantitatively assess the strategy of cells to fill pores and 2) observe the variation in ECM localization and density before and after pore closure.

Macroporous scaffold pore filling proved to be a gradual process driven by the scaffold pore size. Initially, spreading of the cells on the walls was observed and correlated with the presence of fibronectin. When the pore size was in the range of maximal cell spreading length (73.6±22.7 µm), fibroblasts had the possibility of spreading across the pore, which resulted in fibronectin and actin being present already in the middle of some pores after 3 days (Fig. 6a). Assuming cells to behave like tensioned chords allowed to quantitatively describe the process of scaffold pore filling. This model implies that pore filling accelerates towards the end as a consequence of higher curvature in the inner remaining pore diameter (space that was not yet filled by cells) and an accordingly larger area covered by each cell (Fig. 4). The input parameters needed for a simulation of pore closure are only the initial pore geometry and the mean spreading length of the cells and can be measured experimentally. 20-µm stacks were chosen in order to visualize a full interconnected network of cells. Furthermore, going for higher z-values would result in an overlap of cells from different layers when performing the maximum projection. Since the experimental data was compared to a 2D simulation it was important to limit z to a single cell layer Additionally, loss of fibronectin intensity was quickly observed when going further into the pores. Finally, a small z-stack reduced variations in pore shape through the different images of the z-stack.

The geometrical model to simulate scaffold pore filling was based on a chord model developed to understand geometry dependent tissue growth at larger length scales [11]. Cell organization was considered from a geometrical point of view only. For a simulation of the complex cell-cell and cell-matrix interaction cellular mechanical properties like stiffness and traction force generation would have to be taken into account as done by other authors [29]. Realistic models for cell growth would additionally have to consider many parameters such as cell migration, nutrient transport, cell proliferation, and matrix deposition [30]–[32]. Here however, we were interested in the relation between the cell number and the according degree of pore closure. Thus, cell number was the only relevant variable and cell proliferation or nutrient transport issues did not have to be taken into account. Cells were considered as static and, aside from geometry, no interaction between cells was considered. The good agreement between the model and the experimental observations for all pore size categories suggests that the organization of fibroblasts as a consequence of a complex process is defined by simple geometrical constraints. Fibroblasts efficiently fill pores by advancing as far into the open space as their spreading size enables them to do (similar to a tensioned chord). For small pore sizes, spreading across the pores leads to a fast closure. For larger pores, the model predicted an initially slow decrease of the open pore area and an accelerated growth towards the end as a consequence of smaller remaining pore diameter and accordingly higher curvatures. The transition from the process of layer on layer growth to a phase where the cell are able to spread over the remaining pore might have consequences on the ECM structure as a consequence of altered cell arrangement and force distribution in the cellular network.

The model also allowed the introduction of a random factor R considering that the cells might locate randomly or choose positions where they can cover an area as large as possible. In order to further gain insight into the strategy of the cell behavior, a well defined void geometry would be useful. Using the biomaterial, each pore analyzed had indeed a different geometry and initial area to be filled. However, qualitatively, for larger pore, the data suggests that the cells preferentially selected locations of high curvature inside the remaining pore. This could be due to either curvature-dependent migration or proliferation [33]–[34]. Live cell imaging inside the pores would help to further elucidate this aspect. It should be underlined at this point that the model stops when the pore diameter decreased below the cell spreading size. Experimentally, at this final stage of pore filling, cells were observed to behave differently. They adopted a different shape with multiple spatially separated connection points rather than having the typical elongated spindle like appearance as they could not spread anymore to their maximal size.

Fibronectin is one of the early components of the ECM being synthesized and will strongly interact with and regulate the formation and degradation of collagen type I and type III [16]–[18]. Collagen is the most abundant protein in the human body and one of the main components of the ECM structure that, together with elastin, is responsible for the structural framework and mechanical properties of the tissue being formed. Due to the way fibronectin interacts with collagen, the individual mechanical properties of the tissue formed inside the pore will be strongly influenced by the initial fibronectin deposition. It was shown that cells mechanically feel their environment and react to local mechanical stimuli like substrate stiffness and to extrinsic mechanical loading signals [35]. Concerning our study, the behavior of the cells might be potentially affected by the fact that inside the pores, where new tissue has been formed, cells sense a different mechanical environment from what they have been sensed initially when spreading on the walls [36]–[37]. Furthermore the increase in fibronectin network density present in the middle of the pore as suggested by the data in Figure 6a might be a result of ECM compaction due to cellular traction forces. The more tissue forms inside the pore, the higher are the forces on the interface layer with the comparably stiff biomaterial. This might lead to a fibronectin/ECM transport from the periphery to the center of the pore. One has however to be careful as even though the samples were stained in the same way and pictures were taken with the same microscopic settings, antibody diffusion might have been affected by the dense cell and ECM structures inside the pores influencing signal intensity distribution inside the pores. Surprisingly, even though we observed fibronectin to be present everywhere inside the pores of the scaffold after 7 days most of the cells nuclei remain located next to the wall of the scaffold. Further proliferation might have been triggered by changes in local fibronectin density as fibronectin is being pulled from the wall towards the center of the pores. The whole process, from initial spreading of the cells on the pore wall to pore closure and subsequent compaction of the fibronectin network is summarized by a schematic representation in Fig. 7.

For the current study, primary dermal fibroblasts were used as they are known to be key players in the early phase of wound or bone healing [38]–[39]. Their target is to synthesize a specific environment, composed mainly of fibronectin, collagen and elastin that provide first manifestations of structure and mechanical integrity. That first composition is the basis for further tissue maturation and specification by other cells such as MSCs to finally give specific structural properties and mechanical stability to the tissue.

The model presented here has the potential to be adapted to any kind of initial geometry and could be used to predict pore filling for the purpose of scaffold design [40]. Fast tissue growth inside areas of small pores (pores with diameter ≈ cell spreading length) providing early mechanical stabilization could be combined with slow growth inside supply channels (larger pores) that might be developed further into vascular structures. Chiu et al. reported that porous PEG hydrogels could support vascularization, depending also on the pore size [41]. This might have a significant impact in the design of biomaterials [42] to overcome the lack of sufficient vascularization in void healing. A bicomponent macroporous scaffold might be an advantageous solution as suggested by Feng et al. using porous bioceramics [43].

This study was performed in-vitro and under specific conditions of cell and scaffold choice. Consequently, several limitations have to be taken into account and could be the base for further experiments. First of all, tissue deposition is not only realized by fibroblasts. Mesenchymal stromal cells and tissue specific cells (osteoblasts, myoblasts, chondroblasts) are also known to create and remodel their environment. For example MSCs have been shown to stimulate wound healing [44]. Nonetheless, this study is justified by considering that the ratio of MSCs to fibroblasts is quite small and early tissue formation is assumed to be governed by fibroblasts. Also, the initial model and experiments by Bidan et al. were realized using MC3T3-E1 pre-osteoblasts, indicating that this model is not limited to fibroblasts [11]. A second point is that having performed the study under static conditions, the possibility of an insufficient nutrient and oxygen supply might have an impact on the way the pores are being filled even though the scaffold was macroporous [45]. This issue could be addressed by the usage of a bioreactor system to provide a better nutrient supply and compare it to the static case studied here [35]. Another point is that the study was only done using a single type of biohybrid scaffold. The resulting situation might be completely different when using scaffolds having a different surface chemistry, topography or pore architecture. Nevertheless we assume that the general geometric description of pore filling presented here is mostly governed by pore geometry and thus valid for different materials. A lot of evidence has accumulated showing that at least for large pored systems, the generalized chord model and curvature driven growth approach is valid [14], [46]. A premise however is a sufficient mechanical stability of the pore against deformation through cell forces and a proper cell attachment to the biomaterial surface.

## Conclusion

We used here a simple geometric description of the cell organization in scaffold pores that could correctly describe experimental observations of scaffold pore filling by fibroblasts. The model was originally developed to predict tissue growth at a larger scale. We showed here that the number of chords predicted by the adapted model agreed with the number of cells required to fill a scaffold pore. This knowledge might help to understand and finally predict tissue growth in other macroporous biomaterial structures. Only the spreading length of the cell and the initial pore geometry needed to be extracted experimentally to be able to predict void filling. Fibronectin as one component of the extracellular matrix was observed to follow cell organization until pore closure was reached. Once closed, reorganization of fibronectin inside the pore and a compaction of material in the pore center were observed. This might impact subsequent collagen network formation of the tissue inside the pores. These findings allow a better understanding of cell driven pore closure and ECM organization, which is needed for the optimized design of biomaterial scaffold pores for regenerative purposes.

## Supporting Information

Supporting Information S1 **MATLAB code used for the simulations.** The settings for the simulation are given in the structure variable “Settings” (see lines 14–22). The shape of the void is defined by nodes with x- and y-coordinates and saved as complex numbers in P. Iterative adding of “cells” was done in the loop from line 64 to 188. The nodes covered by a “cell” where removed from P for the next step.
(PDF)
