# Supplementary material for: Geometry-Driven Cell Organization Determines Tissue Growths in Scaffold Pores: Consequences for Fibronectin Organization
Source: PLoS One. 2013 Sep 5;8(9):e73545. doi: 10.1371/journal.pone.0073545 (PMC3764044; doi:10.1371/journal.pone.0073545)
Supplement: Supporting Information S1 — MATLAB code used for the simulations. The settings for the simulation are given in the structure variable “Settings” (see lines 14–22). The shape of the void is defined by nodes with x- and y-coordinates and saved as complex numbers in P. Iterative adding of “cells” was done in the loop from line 64 to 188. The nodes covered by a “cell” where removed from P for the next step. (PDF) [file pone.0073545.s001.pdf]

```
function [outX, outY, EVAL] = CellSimulation(Settings)
%% settings
% spl = 80; % µm
% boxA = 400; % µm
% boxB = 400; % µm
% n=[0:0.04:1];
% m=[0:0.04:1];
% Ntotal = 1000;
% AN(1) = boxA*boxB;
% thsld = 0.96; %
%
spl = Settings.spl; % mean spreading length
spl_rnd = Settings.spl_rnd; % max. variation of spreading length
boxA = Settings.xlength; %
boxB = Settings.ylength;
n=[0:0.03:1]; % resolution between nodes (box: 0.01, ellipse: 0.03)
m=[0:0.03:1]; % resolution between nodes (box: 0.01, ellipse: 0.03)
Ntotal = Settings.N; % maximal number of cells (iterations)
thsld = Settings.AreaThrld; % [0..1], strengt of the rule "covering
the biggest area"
%%
EVAL.a(1:Ntotal) = NaN;
EVAL.b(1:Ntotal) = NaN;
EVAL.AN(1) = boxA*boxB; % initial area of rectangular void
EVAL.phi(1:Ntotal) = NaN;
EVAL.X0(1:Ntotal) = NaN;
EVAL.Y0(1:Ntotal) = NaN;
EVAL.long_axis(1:Ntotal) = NaN;
EVAL.short_axis(1:Ntotal) = NaN;
EVAL.status(1:Ntotal) = NaN;
%%
addpath([cd '\fit_ellipse']); % fit_ellipse toolbox (C) 2003, Ohad
Gal
%% define start points (for rectangular void)
P = complex([0 boxA boxA 0],[0 0 boxB boxB]); % initial nodes for
rectangular void
if(strcmp(Settings.Type,'ellipse')), % creates initial nodes for
elliptical void
    el_n = 51;
    el_phi = [360/el_n:360/el_n:360];
    el_a = boxA/2;
    el_b = boxB/2;
    for j=1:length(el_phi)
        asinphi = el_a^2*(sind(el_phi(j))^2);
        bcosphi = el_b^2*(cosd(el_phi(j))^2);
        el_r(j) = el_a*el_b/(sqrt(asinphi + bcosphi));
    end
    el_x = el_r.*cosd(el_phi) + el_a;
    el_y = el_r.*sind(el_phi) + el_b;
    P = complex(el_x,el_y);
end
% sorting P
rad = (2*3.1416)./angle(P-mean(P));
[b,srad] = sort(rad);
P = P(srad);
%
%% start loop
for N=1:Ntotal;
```

```
    % connections between all adjacent Points
    for l=1:length(P)-1
        L(1+(length(n)*(l-1)) : length(n)*l ) = P(l) + n*(P(l+1)-P(l));
    end
    l=l+1;
    L(1+(length(n)*(l-1)) : length(n)*l ) = P(end) + n*(P(1)-P(end));
    % distances between all line points
    [La Lb] = meshgrid(L,L);
    Dist = abs(La-Lb);
    spl_var = round(spl_rnd*rand(1))-Settings.spl_rnd/2; % random variaton of cell spreading length
    mv = min(abs(Dist(:)-spl(:)));
    id = find(abs(Dist-(spl+spl_var)) < mv+1); % finds spl ± 1
    if(~isempty(id))
        [px2,px1] = ind2sub(size(Dist),id);
        A = [];
        inPn = {};
        for k=1:length(px1)
            if(px1(k) < px2(k))
                i = [px1(k):px2(k)];
                i1 = [px1(k)+1:px2(k) px1(k)];
            else
                i = [px1(k):-1:px2(k)];
                i1 = [px1(k)-1:-1:px2(k) px1(k)];
            end
            if(numel(i) > 0.5*numel(L)) % wrong direction, change
                if(px1(k) < px2(k))
                    i = [px2(k):numel(L) 1:px1(k)];
                    i1 = [px2(k)+1:numel(L) 1:px1(k) px2(k)];
                else
                    i = [px1(k):numel(L) 1:px2(k)];
                    i1 = [px1(k)+1:numel(L) 1:px2(k) px1(k)];
                end
            end
            for pi = 1:length(P)
                ppos(pi) = any(abs(P(pi)-L(i))==0);
            end
            inP = P(ppos==1);
            inPn(k) = {find(ppos==1)};
            x = [real(L(px1(k))) real(inP) real(L(px2(k)))];
            x1 = [real(inP) real(L(px2(k))) real(L(px1(k)))];
            y = [imag(L(px1(k))) imag(inP) imag(L(px2(k)))];
            y1 = [imag(inP) imag(L(px2(k))) imag(L(px1(k)))];
            ysum = y+y1;
            xdif = x-x1;
            A(k) = 0.5*abs(sum(ysum.*xdif));
        end
        % select which area will be covered (biggest?)
        [v pmax] = max(A);
        % random effect
        if(thsld<1)
            Athsld = thsld*v;
            Aselect = (v-Athsld)*rand(1)+Athsld;
            [vs, pmax] = min(abs(A-Aselect));
            v = A(pmax);
        end
        % new points/ points to remove
        P1pos = min([px1(pmax) px2(pmax)]);
        P2pos = max([px1(pmax) px2(pmax)]);
        newP1 = L(P1pos);
        newP2 = L(P2pos);
        rmPid = inPn{pmax};
```

```
        if(rmPid>1)
            newPs = [P(1:rmPid-1) newP1 newP2
P(rmPid+1+numel(rmPid)-1:end)];
        elseif(rmPid == 1)
            newPs = [newP1 P(rmPid+1:end) newP2];
        elseif(rmPid == numel(P))
            newPs = [P(1:rmPid-1) newP1 newP2];
        end
        if((any(rmPid==1))&(any(rmPid==numel(P))))
            if(numel(rmPid)<=2)
                newPs = [newP1 P(2:end-1) newP2];
                newPs(rmPid(2:end-1)) = [];
            else
                if( abs(newP1-P(1)) < abs(newP2-P(1)))
                    newPs = [newP1 P(2:end-1) newP2];
                    newPs(rmPid(2:end-1)) = [];
                else
                    newPs = [newP2 P(2:end-1) newP1];
                    newPs(rmPid(2:end-1)) = [];
                end

            end
        end

if((numel(rmPid)>1)&(rmPid(1)==1)&(rmPid(end)==numel(rmPid)))
            if( abs(newP1-P(end)) < abs(newP2-P(end)))
                newPs = [newP1 newP2 P(max(rmPid)+1 : end)];
            else
                newPs = [newP2 newP1 P(max(rmPid)+1 : end)];
            end
        end
        % some evaluation
       % area
        EVAL.AN(N+1) = EVAL.AN(N) - v;
        % area quantification
        ellipse_t = fit_ellipse( real(P),imag(P) );
        if(~isempty(ellipse_t));
            if(~any(strcmp(ellipse_t.status,'Hyperbola found')))
                EVAL.a(N+1) = ellipse_t.a;
                EVAL.b(N+1) = ellipse_t.b;
                EVAL.phi(N+1) = ellipse_t.b;
                EVAL.X0(N+1) = ellipse_t.X0_in;
                EVAL.Y0(N+1) = ellipse_t.Y0_in;
                EVAL.long_axis(N+1) = ellipse_t.long_axis;
                EVAL.short_axis(N+1) = ellipse_t.short_axis;
            else
                continue
            end
        end
        % sorting newPs
        rad = (2*3.1416)./angle(newPs-mean(newPs));
        [b,srad] = sort(rad);
        newPs = newPs(srad);
        % overwrite old nodes with new nodes
        P  = newPs;
    else
        break
    end
    clear ppos inPn L A newPs ellipse_t
end %% end loop
    outX = [0:N-1];
    outY = EVAL.AN/EVAL.AN(1);
end
```
